# Tampering with springs: phosphorylation of titin affecting the mechanical function of cardiomyocytes

**DOI:** 10.1007/s12551-017-0263-9

**Published:** 2017-04-10

**Authors:** Nazha Hamdani, Melissa Herwig, Wolfgang A. Linke

**Affiliations:** 1 Department of Cardiovascular Physiology, Ruhr University Bochum, MA 03/56, 44780 Bochum, Germany; 2 Deutsches Zentrum für Herz-Kreislaufforschung, Partner Site Göttingen, Göttingen, Germany; 3 Cardiac Mechanotransduction Group, Clinic for Cardiology and Pneumology, University Medical Center, Göttingen, Germany

**Keywords:** Cardiomyocytes, Muscle cell mechanics, Posttranslational modification, Heart failure, Diastolic function, Stiffness

## Abstract

Reversible post-translational modifications of various cardiac proteins regulate the mechanical properties of the cardiomyocytes and thus modulate the contractile performance of the heart. The giant protein titin forms a continuous filament network in the sarcomeres of striated muscle cells, where it determines passive tension development and modulates active contraction. These mechanical properties of titin are altered through post-translational modifications, particularly phosphorylation. Titin contains hundreds of potential phosphorylation sites, the functional relevance of which is only beginning to emerge. Here, we provide a state-of-the-art summary of the phosphorylation sites in titin, with a particular focus on the elastic titin spring segment. We discuss how phosphorylation at specific amino acids can reduce or increase the stretch-induced spring force of titin, depending on where the spring region is phosphorylated. We also review which protein kinases phosphorylate titin and how this phosphorylation affects titin-based passive tension in cardiomyocytes. A comprehensive overview is provided of studies that have measured altered titin phosphorylation and titin-based passive tension in myocardial samples from human heart failure patients and animal models of heart disease. As our understanding of the broader implications of phosphorylation in titin progresses, this knowledge could be used to design targeted interventions aimed at reducing pathologically increased titin stiffness in patients with stiff hearts.

**Electronic supplementary material:**

The online version of this article (doi:10.1007/s12551-017-0263-9) contains supplementary material, which is available to authorized users.

## Introduction

Our heart continually adapts to changes in hemodynamic load and responds to neurohumoral stress, which requires the dynamic regulation of cardiac contractile performance on a beat-to-beat basis. Control of heart function is exerted predominantly via signal regulation that often involves transient or longer-lasting post-translational modifications (PTMs) of cardiomyocyte proteins. A frequent and well-studied type of PTM is phosphorylation, which is important to many biological processes (Pawson and Scott 2005) and plays a pivotal role in the regulation of cardiomyocyte protein function (Hamdani et al. 2008a; Solaro 2008). This phosphorylation-mediated regulation is complex and involves compartmentalization and cross-talk between protein kinases (PKs) and protein phosphatases (PPs). Alterations in PK and PP activities are implicated in cardiomyocyte dysfunction and contribute to reduced cardiac output in heart disease. Multiple other PTMs have been found in cardiomyocyte proteins, such as O-GlcNAcylation (Ramirez-Correa et al. 2008), arginylation (Cornachione et al. 2014; Leite et al. 2016; Rai et al. 2008), S-nitrosylation (Figueiredo-Freitas et al. 2015) and oxidation (Alegre-Cebollada et al. 2014; Aryal et al. 2014; Balogh et al. 2014; Beedle et al. 2016; Canton et al. 2011; Eaton et al. 2002; Grützner et al. 2009; Stathopoulou et al. 2016). A myriad of functional consequences have been described for these PTMs, which is an area of intense study. In this review, we focus on PTMs of titin, the largest protein in our body expressed in the contractile units, the sarcomeres.

## The giant protein titin

**Fig. 1:**
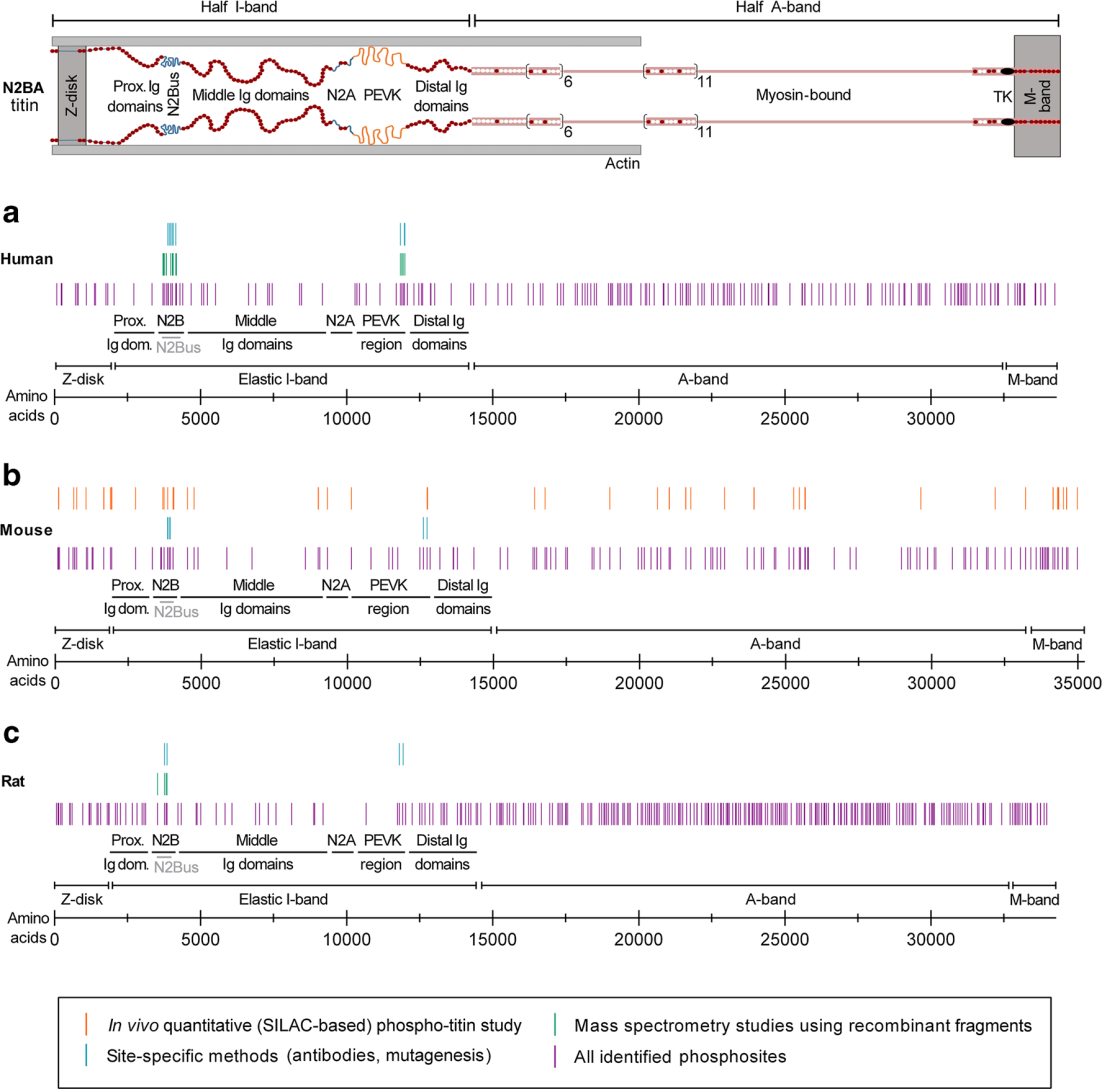
Phosphorylation sites identified in human, mouse, and rat titin. *Top* Layout of the N2BA titin isoform in the cardiac half-sarcomere. **a**–**c** Positions of phosphorylation sites on (**a**) human titin (34,350 amino acids; UniProtKB entry Q8WZ42–1), (**b**) mouse titin (35,213 amino acids; UniProtKB entry A2ASS6.1), and (**c**) rat titin (34,252 amino acids; NCBI entry XP_008773743.1). Only some of the many (potential) phosphosites in titin identified in large phosphoproteomic screens (see www.phosphosite.org; Hornbeck et al. 2015) have been confirmed by site-specific methods such as western blotting with phosphosite-specific antibodies or back-phosphorylation/autoradiography on recombinant wild-type and mutant fragments. One study reported titin phosphosites by in vivo quantitative phosphoproteomics using the SILAC mouse (Hamdani et al. 2013c). A list containing these phosphorylation sites (current as of March, 2017) is provided in the Online Table. *PEVK* titin region rich in proline, glutamate, valine and lysine, *TK* titin kinase domain, *us* unique sequence

The sarcomere is composed of three main filament systems: the myosin-based thick filament, the actin-based thin filament, and the titin filament (Fig. 1, top). Individual titin molecules span the entire 1–2 μm distance of the half-sarcomere from the Z-disk to the M-band. Several alternative splicing events, mostly in the I-band titin region which encompasses more than 220 of the 364 *TTN* exons, produce three main full-length isoforms: shorter, stiffer N2B (3000 kDa) and longer, more compliant N2BA isoforms (>3200 kDa) in the heart, as well as variable-length N2A isoform in skeletal muscles (Bang et al. 2001; Neagoe et al. 2003; Prado et al. 2005). Rare isoforms of titin include the full-length variants Novex-1 and Novex-2, and the truncated Novex-3 isoform (625 kDa). A novel isoform, termed Cronos (2300 kDa), is expressed under the control of an alternative promoter located near the I-band/A-band junction (Zou et al. 2015). Cronos was suggested to have a role in sarcomeric (A-band) assembly, at least in zebrafish (Zou et al. 2015), but a recent study found little evidence to support this hypothesis (Shih et al. 2016). In contrast to the I-band part of titin, almost all exons encoding the A-band and M-band regions of the titin molecule are constitutively expressed. A-band titin is tightly associated with myosin and myosin-binding protein-C (reviewed by Linke and Hamdani 2014). Near the M-band portion of titin is the titin-kinase domain (TK), which was shown to be activated by mechanical strain in vitro (Puchner et al. 2008). However, according to recent structural data, TK may be a pseudokinase (Bogomolovas et al. 2014). Notwithstanding this controversy, TK has been established as a scaffold for multiple protein-protein interactions (Lange et al. 2005; Mayans et al. 1998).

The function of titin as a molecular spring determines the “passive” elasticity of the sarcomere (Li et al. 2002; Linke et al. 1994; Trombitás et al. 1995). The elastic spring region of titin is in the I-band segment of the molecule. This region has a complex structure comprising two types of extensible segments (Freiburg et al. 2000): (1) tandem Ig-domain regions termed ‘proximal-Ig’ (constitutively expressed), ‘middle-Ig’ (alternatively spliced), and ‘distal-Ig’ (constitutively expressed); and (2) intrinsically disordered structures, including the unique sequence of the cardiac-specific ‘N2B’ element (‘N2Bus’) and the ‘PEVK’ segment, which contains numerous 26–28 residue motifs rich in proline, glutamate, valine and lysine (Fig. 1, top). Only repeats 27–31 in the NH_2_-terminal region of the human PEVK segment are constitutively expressed in the full-length titin isoforms. When the cardiac sarcomere is stretched, the Ig-domain segments straighten out before the PEVK and N2Bus elements become extended (Li et al. 2002; Linke et al. 1996; Linke et al. 1999). Sarcomere stretching also increases the unfolding probability of the Ig domains, some of which will unfold at the low passive forces (less than 10 pN/titin molecule) present in sarcomeres that are extended in the physiological working range (Rivas-Pardo et al. 2016). In cardiomyocytes, titin is the primary source of passive tension within a sarcomere length (SL) range of ∼1.8–2.2 μm (Linke et al. 1994) and, thus, is the main determinant of myocardial diastolic passive stiffness during physiological loading (Linke and Hamdani 2014). Apart from being established as the passive elastic spring of the sarcomere, titin has also been implicated in active muscle contraction (Cazorla et al. 2001; Fukuda et al. 2001; Li et al. 2016; Rivas-Pardo et al. 2016).

Titin-based myocardial passive stiffness can be modulated under physiological conditions and in heart disease, including human heart failure (HF). In end-stage failing human hearts, titin-based passive tension is lowered due mainly to isoform switching towards the more compliant N2BA isoform (Neagoe et al. 2002). Another modifier of titin stiffness is the binding of heat shock proteins to elastic I-band domains (Bullard et al. 2004; Kötter et al. 2014). Moreover, titin-based elastic force is affected by PTMs, of which phosphorylation has been studied most intensely and will be covered in detail below. Additional PTMs that alter the stiffness of titin include arginylation (Leite et al. 2016) and various oxidative modifications, such as disulfide bonding (Grützner et al. 2009), S-glutathionylation (Alegre-Cebollada et al. 2014), and sulfenylation (Beedle et al. 2016). The functional role of oxidative modifications in titin has been previously reviewed by us (Beckendorf and Linke 2015; Breitkreuz and Hamdani 2015) and will not be discussed further here. An evolving topic over the last decade has been the pathologically altered titin phosphorylation in HF with preserved or reduced ejection fraction (HFpEF or HFrEF, respectively), which often causes cardiomyocyte stiffening. Global myocardial stiffening is a hallmark of HFpEF (Gladden et al. 2014) and may be due, in part, to dysregulated titin phosphorylation (Linke and Hamdani 2014). Considering the important role of titin phosphorylation for mechanical heart function under physiological and pathological conditions, this review aims to summarize what is known about these properties, with a special focus on the cardiac titin springs.

## Potential and established phosphorylation sites along the titin molecule

In light of its large size, titin could well be the protein with the most phosphorylation sites. Indeed, proteomic databases list hundreds of potential phosphosites in human, mouse, or rat titin (Fig. 1a–c; Supplementary Table), which are searchable by web-based resources, such as http://gygi.med.harvard.edu/phosphomouse/index.php (Huttlin et al. 2010), http://cpr1.sund.ku.dk/cgi-bin/PTM.pl (Lundby et al. 2012), or http://www.phosphosite.org/ (Hornbeck et al. 2015). How phosphorylation affects titin function has been studied for only a limited number of phosphosites. Earlier work demonstrated that proline-directed kinases, including extracellular signal-regulated kinase-1/−2 (ERK1/2) and cyclin-dependent protein kinase-2 (Cdc2), phosphorylate specific motif repeats (*X*SP*X*R; KSP) within the Z-disk and M-band titin regions (Gautel et al. 1993; Gautel et al. 1996; Sebestyen et al. 1995). These phosphorylation events were suggested to be important during developmental stages, e.g., by affecting the interaction of the respective titin region with a binding partner (Fernando et al. 2009). However, little else is known about the functional implications of these PTMs.

A quantitative phosphoproteomics approach using the stable isotope labeling of amino acids (SILAC) mouse compared titin phosphorylation in normal wild-type (WT) mouse hearts with that in mouse hearts deficient in Ca^2+^/calmodulin-dependent protein kinase-II (CaMKII) isoforms γ and δ (Hamdani et al. 2013c). At least 17 different sites along the titin molecule were suggested to be phosphorylated by CaMKII and altogether >70 serine/threonine/tyrosine phosphosites were confirmed in titin (Fig. 1b). Fifteen phosphosites were located in the Z-disk region, 15 in the elastic I-band region, 4 in a region coded by the Novex-3 exon (exon 48), 22 in A-band titin, and 15 in M-band titin. As regards the large unique sequence elements of I-band titin, 7 phosphosites were identified in N2Bus and 4 in the PEVK domain. Importantly, phosphorylation of the N2Bus and PEVK regions has been shown by single-molecule force spectroscopy using atomic force microscopy (AFM) to modify overall titin stiffness (Hidalgo et al. 2009; Krüger et al. 2009; Perkin et al. 2015), as will be discussed further below.

## Phosphosites in titin Ig-domain segments and possible functional role

By in vivo quantitative phosphoproteomics, I-band titin phosphosites were also detected in three Ig domains and in a linker sequence between two such domains, located in the proximal and middle Ig segments and the N2A element, respectively (Hamdani et al. 2013c). Because web-based resources list many more potential phosphosites in the tandem-Ig-domain regions of the titin spring (Fig. 1), one can speculate that at least some of these PTMs affect titin stiffness. Several Ig domains in I-band titin contain phosphosites which are cryptic, i.e., they are buried in the Ig-domain fold and become exposed only after domain unfolding. This is also true for the proximal and middle Ig domains of titin, which preferentially unfold under physiological conditions (Rivas-Pardo et al. 2016). Then, phosphorylation of Ig domains could affect titin stiffness via a mechanism similar to that demonstrated for titin Ig domains that have been oxidized at cryptic cysteines: When the Ig domain is unfolded in the presence of oxidized glutathione (GSSG), refolding is inhibited, and this mechanical weakening causes reduced titin-based cardiomyocyte passive tension (Alegre-Cebollada et al. 2014). If phosphorylation also had such effects, sarcomere stretching could enhance phosphorylation of those Ig domains that have unfolded under the increased stretch force, and thus lower titin-based passive tension. In summary, while phosphorylation of PEVK and N2Bus elements in I-band titin has been established as a modifier of titin elasticity, phosphorylation of Ig-domain segments could also have a mechanical effect on titin and the cardiomyocyte.

## Phosphorylation sites of the cardiac-specific N2Bus element

The N2B element of titin is encoded by *TTN* exon 49 in human and mouse and is expressed specifically in the cardiac isoforms of titin, including N2B and N2BA. Exon 49 codes for three Ig domains and N2Bus (572 residues in human titin). N2Bus can be phosphorylated by several kinases (Linke and Hamdani 2014), including PKA (Krüger and Linke 2006; Yamasaki et al. 2002), cyclic guanosine monophosphate (cGMP) dependent PKG (Krüger et al. 2009), ERK2 (Raskin et al. 2012), and CaMKIIδ (Hamdani et al. 2013c; Hidalgo et al. 2013).

### PKA sites

PKA is activated by cyclic adenosine monophosphate (cAMP) following β-adrenergic stimulation. A PKA-dependent phosphosite in human titin was found at S4185 using back-phosphorylation assays in connection with autoradiography on recombinant WT, deletion and mutation constructs of N2Bus (Krüger et al. 2009). S4185 is present in human N2Bus but not cross-species-conserved. This phosphosite was later verified in a mass-spectrometric screen on PKA-phosphorylated recombinant N2Bus (Kötter et al. 2013). In the same study, additional PKA-dependent phosphosites were detected in human N2Bus at position S4065 (semi-conserved) and in rat N2Bus at S3744 (non-conserved) and S4010 (conserved; this is S3991 in mouse titin), and S4012 (semi-conserved) (Fig. 1a, c). Note that, throughout this review, we refer to the human titin consensus sequence according to UniProtKB entry Q8WZ42–1, the mouse consensus sequence according to UniProtKB entry A2ASS6.1, and the rat consensus sequence according to NCBI entry XP_008773743.1. The complete N2A element and the constitutive part of the PEVK domain were also tested in vitro for PKA-dependent phosphorylation, but were excluded as substrates of this kinase (Krüger et al. 2009). Phospho-specific antibodies were generated against conserved p-S4010 (human)/p-S3991 (mouse) and used to quantify N2Bus phosphorylation by western blot in mouse, dog, or human heart tissue (Hamdani et al. 2013a, b, c; Kötter et al. 2013, 2016; Mohamed et al. 2016; Rain et al. 2014; Tschöpe et al. 2016).

### PKG sites

Cyclic GMP-dependent PKG, which is activated in signaling cascades initiated by nitric oxide (NO) or natriuretic peptides, also phosphorylates N2Bus. The first PKG-dependent phosphosite described for titin (using site-directed mutagenesis and back-phosphorylation of recombinant human N2Bus) was S4185 (non-conserved), which is also phosphorylated by PKA (Krüger et al. 2009). Additional PKG-dependent phosphosites were identified by mass-spectrometry of recombinant human N2Bus at S4092 (semi-conserved) and S4099 (conserved; this is S4080 in mouse titin) (Kötter et al. 2013). In recombinantly expressed rat N2Bus, S3744 (non-conserved) was detected as a PKG-phosphorylated site. PKG did not phosphorylate the constitutive part of the human PEVK domain in vitro, but, interestingly, it did phosphorylate the N2A element of I-band titin (Krüger et al. 2009). Phospho-specific antibodies made against p-S4099 (human)/p-S4080 (mouse) and p-S4185 (human) have served to quantify N2Bus phosphorylation in mouse, dog, or human heart tissue (Eisenberg et al. 2016; Hamdani et al. 2013a, c; Kötter et al. 2013, 2016; Mohamed et al. 2016; Müller et al. 2014; Rain et al. 2014; Zile et al. 2015).

### ERK2 sites

ERK2 is an effector kinase of the mitogen-activated protein kinase (MAPK) pathway activated by upstream kinase Raf1. ERK2 is recruited to N2Bus, together with two of its upstream MAPKs, via four-and-a-half LIM-domain-1 (FHL-1) protein (Sheikh et al. 2008). ERK2 phosphorylates N2Bus at specific sites, as detected using back-phosphorylation assays on recombinant WT and mutated constructs of human N2Bus (Raskin et al. 2012). The following phosphosites were found to be ERK2-dependent (reference to the human titin consensus sequence): S3918 and S3960 (both non-conserved), and S4010 (conserved). Thus, S4010 (=S3991 in mouse titin) is phosphorylated by both ERK2 and PKA. Notably, the N2Bus-binding partner FHL-1 blocked phosphorylation of N2Bus at several residues in vitro, including S4010 (Raskin et al. 2012). However, because phospho-S4010 is well recognized by phospho-specific antibodies in heart tissue (Hamdani et al. 2013b, c; Kötter et al. 2013, 2016; Mohamed et al. 2016), the blocking may be ineffective in vivo.

### CaMKII sites

The predominant CaMKII isoform in the heart is CaMKIIδ, which phosphorylates N2Bus at multiple sites (Hamdani et al. 2013c; Hidalgo et al. 2013). Using the above-mentioned SILAC mouse model, quantitative titin phosphoproteomics were performed comparing heart tissue from WT and CaMKIIγ/δ double-knockout (KO) mice (Hamdani et al. 2013c). The CaMKII-dependent phosphosites thus detected in N2Bus were mostly non-conserved residues. However, a conserved phospho-serine at S4062 (in human titin/S4043 in mouse titin) reacted with phospho-specific antibodies directed at this phosphosite, and the observed hypo-phosphorylation at this position in CaMKIIγ/δ double-KO mouse hearts suggested it is a preferred CaMKII phosphosite (Hamdani et al. 2013c). This finding was further supported by hyper-phosphorylation of p-S4043 in a transgenic mouse model overexpressing CaMKIIδ (Hamdani et al. 2013c). In a parallel study, recombinantly expressed human N2Bus was phosphorylated by CaMKIIδ and phospho-residues were identified by mass spectrometry (Hidalgo et al. 2013). Various non-conserved serines and threonines in N2Bus were found to be CaMKIIδ-dependent, while two phosphosites were highly conserved (S3750 and S4209).

Taken together, the mechanically active N2Bus element in cardiac titin has been established as a hot spot for phosphorylation by different PKs. At the same time, little is known about protein phosphatases that dephosphorylate N2Bus, although PP1, PP2a or alkaline phosphatase have been used experimentally to dephosphorylate titin in vitro (Hidalgo et al. 2009; Krüger and Linke 2006; Krüger et al. 2009). It will be interesting to determine which PP(s) dephosphorylate(s) N2Bus under physiological conditions in cardiomyocytes.

## Phosphorylation of the N2A element

The recombinant N2A region of titin consisting of four Ig domains and a longer unique sequence insertion was phosphorylated by PKG in back-phosphorylation assays (Krüger et al. 2009). However, the precise phosphorylation sites were not determined. Notably, phosphorylation by PKG did not appear to alter the mechanical properties of the N2A element in single-molecule AFM force-extension measurements (Krüger et al. 2009). Catalytic subunit of PKA was also used in phosphorylation tests on recombinant N2A, but it did not phosphorylate this titin region (Krüger et al. 2009). In contrast, in another study, PKA did phosphorylate the recombinant unique sequence insertion of N2A encompassing 134 residues (Lun et al. 2014). A binding partner of this titin region, ankyrin repeat domain (Ankrd) protein (Miller et al. 2003), partially blocked PKA-mediated phosphorylation of the unique sequence insertion in vitro, an effect shown for both Ankrd1 and Ankrd23 proteins (Lun et al. 2014). The functional implications of these modifications remain unknown, but could be related to the putative mechanosensor function of the N2A element (Linke 2008; Lun et al. 2014).

## Phosphorylation sites of the PEVK domain

Studies of site-specific phosphorylation in PEVK, another important spring element in I-band titin (Linke et al. 1998), have focused exclusively on the relatively short COOH-terminal portion coded (in human titin) by exons 219–223. This is the ∼185-residue-long part of PEVK that is constitutively expressed in the full-length isoforms, including N2B (where it is the only bit of PEVK expressed), N2BA, and N2A in skeletal muscle (Freiburg et al. 2000). The remainder of the PEVK element (differentially spliced) contains up to ∼2000 residues and has several potential phosphorylation sites (Fig. 1), which still await verification by site-specific methods. As of now, two different PKs have been reported to phosphorylate the constitutively expressed PEVK in cardiac muscle, PKCα (Hidalgo et al. 2009) and CaMKIIδ (Hamdani et al. 2013c; Hidalgo et al. 2013).

### PKCα sites

PKC is activated by the α_1_-adrenergic signaling pathway and PKCα, the predominant isozyme in the heart, is a key player in contractile dysfunction and HF (Hamdani et al. 2008a). PKCα phosphorylated recombinant human PEVK domain at two conserved serines, S11878 and S12022, as determined by mass spectrometry in combination with site-directed mutagenesis and back-phosphorylation assays (Hidalgo et al. 2009). Treatment of the PEVK fragment with protein phosphatase-1, prior to phosphorylation by PKCα, exacerbated the effect. The cardiac N2Bus element was not phosphorylated by PKCα in vitro. When the constitutively expressed PEVK region was deleted in mouse hearts, PKCα phosphorylation was abolished (Hudson et al. 2010).

### CaMKIIδ sites

Multiple lines of evidence have documented PEVK phosphorylation by CaMKIIδ in vivo and in vitro and may have provided the strongest data on site-specific titin phosphorylation yet available. SILAC-based quantitative phosphoproteomics using WT and CaMKIIγ/δ double-KO mouse hearts identified CaMKII-mediated phosphorylation at three conserved PEVK sites, T12869, S12871, and S12884, which correspond to T12007, S12009, and S12022 in human titin, respectively (Hamdani et al. 2013c). These phospho-residues were verified in site-directed mutagenesis/back-phosphorylation experiments with recombinant PEVK to be CaMKIIδ-dependent (Hamdani et al. 2013c). A mass-spectrometric screen of recombinant PEVK also found CaMKIIδ phosphorylation at S12022 and suggested that S11878 (S12742 in mouse titin) may also be phosphorylated by this kinase (Hidalgo et al. 2013); however, the latter was not confirmed elsewhere (Hamdani et al. 2013c). Three non-conserved residues, T11922, T11932 and T11969, were detected in vitro as additional CaMKIIδ-dependent phosphosites (Hidalgo et al. 2013).

Phospho-specific antibodies identified unaltered phosphorylation at S12742 in CaMKIIγ/δ double-KO versus WT mouse hearts, but significantly reduced phosphorylation at S12884 (Hamdani et al. 2013c). Thus, at least S12884 (mouse)/S12022 (human) can be phosphorylated by both CaMKIIδ and PKCα. The phospho-specific antibodies against p-S11878 and p-S12022 (p-S12742 and p-S12884 in mouse titin) have been used repeatedly to quantify PEVK phosphorylation by western blot in mouse, rat, dog, or human hearts (Hamdani et al. 2013a, b, c; Hidalgo et al. 2014; Hudson et al. 2011; Hutchinson et al. 2015; Kötter et al. 2013, 2016; Kovács et al. 2016; Mohamed et al. 2016; Rain et al. 2014; Tschöpe et al. 2016; Zile et al. 2015).

Taken together, the constitutively expressed portion of PEVK is a second hot spot for phosphorylation in I-band titin. As with some phosphosites in N2Bus, a number of conserved residues in PEVK can be phosphorylated by more than a single PK. While it remains unknown which PPs dephosphorylate PEVK physiologically, PP1 is a good candidate, as it dephosphorylated the PEVK element in vitro.

## Effects of titin segment phosphorylation on stretch-dependent titin spring force

Phosphorylation of the unique elements in the titin spring segment has a mechanical effect on the sarcomere. This was initially shown for PKA-mediated phosphorylation of N2Bus, which reduced the passive tension of skinned rat and bovine cardiomyocytes (Yamasaki et al. 2002; Fukuda et al. 2005). The effect was confirmed in single rat cardiac myofibrils and was also shown to occur in human cardiac fibers, but not skeletal myofibers (Krüger and Linke 2006). Even a single cardiomyocyte contains many different structural elements, including cytoskeletal filaments, which potentially contribute to passive tension and elasticity (Robinson et al. 2016). Therefore, the most direct evidence for the effect of titin phosphorylation on titin spring force has come from single-molecule mechanical measurements using AFM force spectroscopy (Krüger et al. 2009; Hidalgo et al. 2009).

Mechanical manipulation of single human N2Bus molecules (flanked by Ig domains) in AFM force-extension experiments showed that the presence of cGMP-activated PKG increased the persistence length, an important parameter of (entropic) polymer elasticity and indicator of the polymer’s bending rigidity, by a factor of ∼2 (Krüger et al. 2009). Such an alteration in polymer elastic properties is predicted to lower the force needed to stretch the polymer to a given length. Using the wormlike chain model, the increase in persistence length of N2Bus was predicted to lower the force of the whole cardiac titin spring segment by nearly 20% (Krüger et al. 2009). In an earlier pilot study, phosphorylation of N2Bus by PKA surprisingly had no significant effect on single-molecule N2Bus elasticity, as judged by unaltered persistence length (Leake et al. 2006). In contrast, both ERK2 and CaMKIIδ increased the persistence length of N2Bus in AFM force-extension measurements by a factor of 2–3 and thus lowered the single-molecule force (Perkin et al. 2015). The effect of CaMKIIδ on force reduction was larger than that of ERK2. In summary, phosphorylation of N2Bus increases the persistence length, which causes reduced overall titin stiffness and explains a modest decrease in passive tension of a cardiac sarcomere or cardiomyocyte.

The mechanical effect of phosphorylation by PKCα on the constitutively expressed PEVK domain was also probed in a single-molecule AFM force-extension study (Hidalgo et al. 2009). Other than with N2Bus, phosphorylation reduced the persistence length of PEVK and increased the stretch-dependent force of the titin spring, thus elevating cardiomyocyte passive tension. The increase was on the order of 20–30%. Taken together, the molecular stiffness of the N2Bus and PEVK elements is changed in opposite directions upon phosphorylation. Phosphorylation of N2Bus reduces overall titin stiffness, phosphorylation of PEVK increases it.

A possible explanation for this opposing effect is the different net charge of N2Bus and PEVK (Kötter et al. 2013; Linke and Hamdani 2014). N2Bus contains many acidic residues, accounting for the low isoelectric point of this domain. In contrast, the constitutively expressed part of PEVK has many basic residues and a much higher isoelectric point. Introducing negatively charged phosphate groups into the negatively charged environment of N2Bus could increase intramolecular electrostatic repulsion and lower the compactness of the intrinsically disordered N2Bus, thereby increasing its distensibility and reducing the force at a given extension. Conversely, addition of negatively charged phosphate residues into the positively charged environment of PEVK would promote electrostatic attraction, lower distensibility, and increase the force at a given extension. According to this theory, phosphorylation of elastic titin regions by different kinases can produce different mechanical effects, depending on *where* the PK phosphorylates the titin spring.

## Changes in cardiomyocyte stiffness upon phosphorylation by different kinases

Various studies on myocardial samples of different species have provided evidence that those PKs that predominately phosphorylate N2Bus usually lower the passive tension of cardiomyocytes, whereas those phosphorylating PEVK typically increase it. PKA-mediated phosphorylation (specifically, ex-vivo treatment with the catalytic subunit of PKA) caused a reduction in passive tension of permeabilized cardiomyocytes isolated from human (Borbély et al. 2009; Falcão-Pires et al. 2011; Krüger and Linke 2006; Rain et al. 2014; van Heerebeek et al. 2006), rat (Fukuda et al. 2005; Yamasaki et al. 2002), cow (Fukuda et al. 2005), mouse (Hamdani et al. 2008b), and dog heart (Hamdani et al. 2013a). PKG-dependent phosphorylation also resulted in reduced passive tension, as demonstrated for human (Borbély et al. 2009; Krüger et al. 2009), rat (Hamdani et al. 2013b), and dog cardiomyocytes (Hamdani et al. 2013a). Likewise, ERK2-mediated phosphorylation decreased the passive tension of mouse papillary muscles (Perkin et al. 2015). However, PKCα-mediated phosphorylation caused increased passive tension in myocardial strips from mouse and pig hearts (Hidalgo et al. 2009) and in cardiomyocytes from human heart (Rain et al. 2014).

As CaMKIIδ phosphorylates both N2Bus and PEVK, a neutral effect of the kinase on passive tension could perhaps be expected. However, the passive force of skinned mouse cardiomyocytes was lowered by CaMKIIδ-treatment (Hamdani et al. 2013c) and at least a trend for reduction was seen in skinned human cardiomyocytes (Rain et al. 2014). In support of these findings, a substantial (∼30%) reduction in passive tension occurred upon incubation of mouse papillary muscles with CaMKIIδ (Perkin et al. 2015). Furthermore, cardiomyocytes of CaMKIIγ/δ double-KO mice had increased passive tension, whereas those of CaMKIIδ-overexpressing transgenic mice had reduced passive tension, compared to those of WT mice (Hamdani et al. 2013c). Therefore, the mechanical effect on N2Bus may dominate over that on PEVK. We thus conclude that CaMKII usually lowers titin-based spring force. Hence only one kinase (PKCα) has been identified yet, which increases titin-based stiffness, whereas four kinases (PKA, PKG, ERK2 and CaMKIIδ) are known to reduce this stiffness.

## Alterations of titin phosphorylation in heart failure

Evidence from our and other laboratories has shown that pathologically altered titin phosphorylation can occur in failing hearts of human patients and animal models, but can also be rescued ex vivo and in vivo (Linke and Hamdani 2014).

### All-titin phosphorylation in human hearts

This parameter has been measured by using the ProQ Diamond (phosphoprotein)/Sypro Ruby (total protein) dual-staining system, western blotting with anti-phosphoserine/threonine antibodies, or autoradiography after back-phosphorylation assays. Using the latter, a deficit for PKG-dependent phosphorylation of titin was detected in a pioneering study on end-stage failing human hearts from patients with dilated cardiomyopathy (DCM) compared to non-failing donor hearts (Krüger et al. 2009). In LV endomyocardial biopsies from patients with HFrEF, aortic stenosis, or HFpEF, increased ratios of phospho-N2BA:phospho-N2B were found by ProQ Diamond/Sypro Ruby staining (Borbély et al. 2009; Falcão-Pires et al. 2011), although this may not necessarily indicate altered phosphorylation per se, considering that titin isoforms switch towards N2BA in failing human hearts (Neagoe et al. 2002; Schafer et al. 2017). Furthermore, total-titin phosphorylation (by ProQ Diamond/Sypro Ruby staining) was unaltered in another set of explanted human DCM hearts, while hypertrophic cardiomyopathy (HCM) hearts showed modest hypo-phosphorylation of titin (Kötter et al. 2013). In right ventricular (RV) samples from patients with pulmonary arterial hypertension, all-titin phosphorylation (by ProQ Diamond/Sypro Ruby staining) was lowered compared to control samples from humans with normal pulmonary pressures (Rain et al. 2013). In a small case study, total-titin phosphorylation (by ProQ Diamond/Sypro Ruby staining) was increased in cardiac biopsies of an HFpEF patient following delivery of electrical signals during the absolute refractory period (cardiac contractility modulation), which aims to improve contraction (Tschöpe et al. 2016). These findings suggested that all-titin phosphorylation is increased in some forms of human HF, but decreased or unaltered in other forms. Results also provided evidence for reversibility of the titin phosphorylation changes that occur in failing hearts.

### All-titin phosphorylation in myocardium from animal models

In dog and rat models of HFpEF versus healthy animal hearts, all-titin phosphorylation (by ProQ Diamond/Sypro Ruby staining) was reduced, but could be normalized by ex vivo treatment with PKG (Hamdani et al. 2013a; Hamdani et al. 2013b). Moreover, in vivo administration of the cGMP-enhancing agents, sildenafil and brain natriuretic peptide, acutely increased total-titin phosphorylation in the dog model (Bishu et al. 2011). A more recent study on the dog HFpEF model, however, found increased total-titin phosphorylation (by ProQ Diamond/Sypro Ruby staining) in several cardiac chambers, compared to normal dog hearts (Zakeri et al. 2016). In mouse hearts with diastolic dysfunction due to experimental transverse aortic constriction, PKA-mediated total titin phosphorylation measured via back-phosphorylation/autoradiography was increased (suggesting lowered titin-based stiffness), but myocardial passive tension was higher than in controls (Hudson et al. 2011). Several other experimental mouse models have been studied for alterations in total cardiac titin phosphorylation. In mice exposed to acute or chronic volume overload, total-titin phosphorylation (by anti-phosphoserine/threonine antibodies on western blots) was significantly reduced, compared to sham-operated animals (Mohamed et al. 2016). This finding differed from that of a study on a similar mouse model, in which no alterations were detected in PKA-mediated total-titin phosphorylation (via back-phosphorylation/autoradiography) (Hutchinson et al. 2015). Furthermore, in a murine model of myocarditis, titin phosphorylation (by ProQ Diamond/SyproRuby stain) was significantly reduced but could be restored to normal levels following virus-mediated injection of an inhibitor of the interleukin-6 receptor (Savvatis et al. 2014). Along the same line, the reduction in total-titin phosphorylation (by ProQ Diamond/SyproRuby stain) and increase in cardiomyocyte passive tension observed in obese type-2 diabetic versus healthy mice—which may be related to pathological insulin signaling affecting titin properties (Krüger et al. 2010; Falcão-Pires et al. 2011)—were reversed by oral application of sitagliptin-4, a dipeptidyl peptidase-4 inhibitor prescribed to diabetic patients (Hamdani et al. 2014). Moreover, in aging mouse and hypertensive rat hearts, all-titin phosphorylation could be increased by oral administration of a natural polyamine, spermidine, which is known, among others, to increase NO bioavailability and hence promote cGMP-PKG signaling (Eisenberg et al. 2016). Many of these studies have thus shown that cardiac titin phosphorylation is malleable and can be manipulated by various interventions.

In summary, failing versus healthy human and animal heart tissue samples have been studied extensively for all-titin phosphorylation. Findings have provided initial hints at changes in titin phosphorylation in diverse pathologies, as well as reversal of phosphorylation through specific treatments. However, it needs to be acknowledged that measuring all-titin phosphorylation provides only limited insight, considering the hundreds of (potential) phosphosites present in titin (Fig. 1).

### Site-specific titin phosphorylation in human hearts

In attempts to overcome this limitation, the phospho-specific antibodies generated against N2Bus and PEVK phosphosites have been used to obtain more detailed information on the phosphorylation status of the unique spring elements of titin in heart disease. For HCM and DCM human end-stage failing versus donor hearts, a study found hypo-phosphorylation of N2Bus at PKA/ERK2 site p-S4010 and PKG sites p-S4099 and p-S4185 (Kötter et al. 2013). Conversely, PKCα-dependent phosphosite p-S11878 in PEVK was hyper-phosphorylated. Both these alterations are anticipated to increase titin-based passive tension, and this was indeed observed in isolated myocardial strips from failing compared to donor hearts (Kötter et al. 2013). In another study, cardiac titin was hypo-phosphorylated at S4185 (PKG/PKA site in N2Bus) but hyper-phosphorylated at S11878 (PKCα site in PEVK) in HFpEF patients with hypertension, compared to non-hypertensive HFpEF patients and control subjects (Zile et al. 2015). No significant change was detected at p-S12022 (CaMKIIδ and PKCα site in PEVK). The alterations in N2Bus and PEVK phosphorylation were associated with increased titin-based stiffness. Moreover, in a subset of end-stage failing patient hearts showing increased CaMKIIδ expression and activity, N2Bus sites p-S4010 (PKA/ERK2-dependent) and p-S4062 (CaMKIIδ-dependent) were hyper-phosphorylated compared to non-failing donor hearts, as was PEVK site p-S11878 (PKCα-dependent), whereas p-S12022 (CaMKIIδ/PKCα-dependent) showed a trend for increased phosphorylation and p-S4099 (PKG-dependent) remained unaltered (Hamdani et al. 2013c). In RV samples from patients with pulmonary arterial hypertension, N2Bus phosphorylation at p-S4185 was lower than in non-failing donor hearts, as was PEVK phosphorylation at p-S12022, whereas p-S11878 was unaltered (Rain et al. 2014). These changes were suggested to determine the increased RV cardiomyocyte passive tension of the patients.

### Site-specific titin phosphorylation in hearts of experimental animals

Animal models of heart disease largely recapitulated the changes in cardiac N2Bus/PEVK phosphorylation seen in human patients. In a dog model of early HFpEF, a phosphorylation deficit was found at N2Bus sites S4010 (PKA/ERK2) and S4099 (PKG), whereas phosphorylation at PEVK site S11878 (PKCα) was increased (Hamdani et al. 2013a). In a metabolic risk-induced animal model of HFpEF, the obese Zucker spontaneously hypertensive fatty-1 (ZSF1) rat, N2Bus phosphorylation at S4010 (S3991 in rat) was lower, but PEVK phosphorylation at S12022 (S12884 in rat) was higher than in healthy rat hearts, whereas PEVK phosphorylation at S11878 (S12742 in rat) was unaltered (Hamdani et al. 2013b). The hearts of renin-overexpressing, hypertensive rats showed increased phosphorylation at PEVK site S12742 and unaltered phosphorylation at S12884, while N2Bus phosphosites were not studied (Kovács et al. 2016). In mouse hearts stressed by pressure overload due to transverse aortic constriction surgery, PEVK site p-S11878 (PKCα) was hyper-phosphorylated and PEVK site p-S12022 (CaMKIIδ/PKCα) hypo-phosphorylated, whereas N2Bus phosphosites were again not studied (Hudson et al. 2011). In murine hearts exposed to acute or chronic volume overload, hypo-phosphorylation was found at N2Bus sites p-S3991 and p-S4080 (p-S4043 was unaltered) but also at PEVK site p-S12884, while PEVK site p-S12742 was hyper-phosphorylated, compared to sham-operated control hearts (Mohamed et al. 2016). These changes suggested a stiffer titin. Surprisingly, a similar mouse model showed no alterations in site-specific phosphorylation at S12742 and S12022, but N2Bus sites were not studied (Hutchinson et al. 2015). In a different experimental mouse model, cardiomyocyte stiffening was seen in early adaptive ventricular remodeling following myocardial infarction and was explained by altered site-specific titin phosphorylation, as PEVK phosphorylation at S11878 and S12022 was increased, whereas N2Bus phosphorylation at S4010 and S4099 was initially unaltered but later became reduced (Kötter et al. 2016). Finally, a dramatic, beneficial, reduction in myocardial diastolic stiffness was achieved in aging mouse or hypertensive rat hearts by oral administration of spermidine, and this effect was associated with increased titin phosphorylation at PKG-dependent N2Bus site p-S4080 (Eisenberg et al. 2016).

Interestingly, altered titin phosphorylation also occurred in rodent hearts in response to physiological exercise. Treadmill running caused altered site-specific cardiac titin phosphorylation in rats, compared to sedentary controls: N2Bus site p-S4099 (rat S4080) was hypo-phosphorylated and p-S4010 (rat S3991) unaltered, while PEVK site p-S11878 (rat S12742) was hyper-phosphorylated and p-S12022 (rat S12884) hypo-phosphorylated (Müller et al. 2014). In a similar study on mice, p-S12022 again responded to exercise with reduced phosphorylation, while p-S11878 was unaltered (Hidalgo et al. 2014). These changes were expected to lower cardiac titin stiffness.

## Relevance of altered titin phosphorylation for cardiac function

Taken together, a general picture has emerged according to which failing human and animal hearts typically (but not always) show hypo-phosphorylation at N2Bus sites and hyper-phosphorylation at PEVK sites. These differential changes are predicted to coordinately increase titin-based passive tension in HF, including HFpEF. Reversal of the pathological titin phosphorylation pattern may be achieved by exercise training or specific drug interventions and appears to be helpful in reducing the pathologically increased myocardial stiffness in disease. Some of the above findings imply that the differential phosphorylation of the elastic titin elements depends on the up- or down-regulation of kinase signaling pathways (Linke and Hamdani 2014). Pharmacological targeting of these pathways could be useful in treating HF patients with a stiff heart.

A relevant question that arises out of these observations is whether or not reduced phosphorylation of N2Bus sites and increased phosphorylation of PEVK sites should generally be considered pathological, because they both increase titin-based stiffness. Increased myocardial passive stiffness is a key alteration seen in the majority of HFpEF patients (Zile et al. 2004) and is considered detrimental to cardiac function. The differential changes in titin phosphorylation at N2Bus and PEVK sites observed in failing human and animal hearts are thus likely to promote diastolic dysfunction. However, increased titin-based stiffness may also have beneficial effects on the heart. There is now excellent evidence suggesting that lowered titin-based stiffness is associated with reduced length-dependent activation (LDA) of the contractile apparatus (Ait-Mou et al. 2016; Beqqali et al. 2016; Cazorla et al. 2001; Fukuda et al. 2001, 2003; Li et al. 2016; Methawasin et al. 2014; Patel et al. 2012; Terui et al. 2008). LDA is the molecular basis for the Frank–Starling relationship, a long-accepted law of heart function stating that increased diastolic filling causes increased contractility. Moreover, if titin stiffness is raised experimentally due to deletion of the N2B region, LDA is enhanced (Lee et al. 2010). Therefore, one can expect that the increased titin-based stiffness that follows from the differential changes in N2Bus and PEVK phosphorylation also promotes the Frank–Starling mechanism. The alterations in titin phosphorylation observed in heart failure could thus have a beneficial effect on systolic pump function, perhaps acting as a compensatory mechanism that helps mobilize the contractile reserve of the failing heart.

In conclusion, considerable progress has been made in recent years in understanding how phosphorylation modifies titin, cardiomyocyte, and global myocardial mechanical function. Nevertheless, the functional relevance of most of the phosphosites in titin remains to be discovered. As our knowledge of the broader implications of phosphorylation in titin progresses, new therapeutic opportunities may become apparent whereby targeted interventions to reduce titin stiffness can be used to correct diastolic LV dysfunction and improve the outcomes of HF patients with diastolic dysfunction.

## Electronic supplementary material

ESM 1 (XLSX 70 kb)
